# Hyperbaric Normoxia Improved Glucose Metabolism and Decreased Inflammation in Obese Diabetic Rat

**DOI:** 10.1155/2019/2694215

**Published:** 2019-11-19

**Authors:** Naoto Fujita, Natsuki Goto, Tomoya Nakamura, Wataru Nino, Taketoshi Ono, Hisao Nishijo, Susumu Urakawa

**Affiliations:** ^1^Department of Musculoskeletal Functional Research and Regeneration, Graduate School of Biomedicine and Health Sciences, Hiroshima University, 1-2-3 Kasumi, Minami-ku, Hiroshima 734-8553, Japan; ^2^System Emotional Science, Graduate School of Medicine and Pharmaceutical Sciences, University of Toyama, Sugitani 2630, Toyama 930-0194, Japan

## Abstract

Hyperbaric treatment improves hyperglycemia and hyperinsulinemia in type 2 diabetes associated with obesity. However, its mode of action is unknown. The purpose of the present study was to investigate the influences of regular hyperbaric treatment with normal air at 1.3 atmospheres absolute (ATA) on glucose tolerance in type 2 diabetes with obesity. The focus was directed on inflammatory cytokines in the adipose tissue and skeletal muscle. Otsuka Long-Evans Tokushima Fatty (OLETF) rats were used as models of type 2 diabetes with obesity and Long-Evans Tokushima Otsuka (LETO) rats served as healthy controls. The rats were randomly assigned to untreated or hyperbaric treatment groups exposed to 1.3 ATA for 8 h d^−1^ and 5 d wk^−1^ for 16 wks. Glucose levels were significantly higher in the diabetic than in the healthy control rats. Nevertheless, glucose levels at 30 and 60 min after glucose administration were significantly lower in the diabetic rats treated with 1.3 ATA than in the untreated diabetic rats. Insulin levels at fasting and 120 min after glucose administration were significantly lower in the diabetic rats treated with 1.3 ATA than in the untreated diabetic rats. Hyperbaric treatment also increased interleukin-10 (IL-10) expression in the skeletal muscle and decreased tumor necrosis factor *α* (TNF*α*) expression in adipose tissue. These results suggested that TNF*α* downregulation and IL-10 upregulation in diabetic rats subjected to hyperbaric treatment participate in the crosstalk between the adipose and skeletal muscle tissues and improve glucose intolerance.

## 1. Introduction

Nutrient overload induces low-grade chronic inflammation of the adipose tissue, skeletal muscle, liver, pancreas, and hypothalamus. Insulin resistance and glucose intolerance develop as a consequence of this inflammation [1]. Insulin receptors and inflammatory signaling interact at the insulin receptor substrate level. Proinflammatory cytokines released from adipose and other inflammatory tissues interfere with glucose uptake via the insulin signaling pathway [2]. It is now recognized that inflammation associated with chronic obesity leads to type 2 diabetes [3].

Medication [4], diet [5], and exercise [6] are the key therapeutic approaches to the management of type 2 diabetes with obesity. They improve insulin resistance and glucose intolerance and reduce inflammation. Previous studies have demonstrated the efficacy of anti-inflammatory therapy and other strategies targeting proinflammatory cytokines such as interleukin-6 (IL-6) [7], tumor necrosis factor *α* (TNF*α*) [8], and interleukin-1*β* (IL-1*β*) [9]. Medication therapy is a critical component of the treatment of patients with obese diabetes. However, this approach is costly. Therefore, the application of a low-cost complementary treatment such as physical exercise is recommended for the clinical management of this disorder.

The benefits of regular physical exercise for the treatment of patients with type 2 diabetes and obesity are widely known. The American College of Sports Medicine recommends regular aerobic exercise and resistance training to improve insulin resistance and glucose intolerance in diabetic patients [10]. We confirmed the efficacy of physical exercise in a rat model of type 2 diabetes with obesity [11]. As many patients dislike physical activity, however, this therapeutic approach is often inconsistently practiced.

It was reported that hyperbaric treatment stimulates the skeletal muscle and improves insulin resistance and glucose intolerance. It maintains elevated atmospheric pressure and oxygen concentration with an air compressor and an oxygen concentrator. It enhances the partial pressure of oxygen and increases the concentration of dissolved oxygen in the blood [12]. We confirmed the efficacy of hyperbaric treatment in type 2 diabetes. This therapy increases oxidative metabolism in the skeletal muscles [13–15]. Hyperbaric treatment requires no physical activity on the part of patients with type 2 diabetes and obesity. Therefore, they may be more likely to submit to it. Recently, hyperbaric treatment with normal air at <2 atmospheres absolute (ATA) has been substituted for concentrated oxygen and is gaining popularity as a home remedy. However, it remains to be determined whether hyperbaric treatment using normal air at <2 ATA is as effective at improving obese type 2 diabetes as concentrated oxygen. Moreover, the effects of hyperbaric treatment on inflammatory cytokines in type 2 diabetes with obesity remain unknown. Hyperbaric treatment using normal air is usually performed at 1.3 ATA, and this condition is now very common for the convenience of users. The purpose of the present study was to investigate the influences of regular hyperbaric treatment using normal air at 1.3 ATA on glucose tolerance in type 2 diabetes with obesity. This research also focused on inflammatory cytokines in the adipose tissue and skeletal muscle.

## 2. Materials and Methods

### 2.1. Experimental Design

This study was approved by the Institutional Animal Care and Use Committee of Hiroshima University (A16-5) and conducted in accordance with the Hiroshima University Regulations for Animal Experimentation. All experiments complied with the National Institute of Health Guidelines for the Care and Use of Laboratory Animals.

Male Otsuka Long-Evans Tokushima Fatty (OLETF) rats (*n* = 12) were used as models of type 2 diabetes with obesity. Diabetic rats aged 24 wks were randomly assigned either to an untreated (Diabetes, *n* = 6) or a hyperbaric treatment (Diabetes HB, *n* = 6) group. The Diabetes group was further divided into subgroups according to the body weights (Diabetes obesity, *n* = 4; Diabetes lean, *n* = 2). In the Diabetes obesity group, the body weights were higher at the end of the experiment than at the start of the experiment. Conversely, in the Diabetes lean group, the body weights were lower at the end of the experiment than at the start of the experiment. Age-matched male Long-Evans Tokushima Otsuka (LETO) rats (*n* = 12) were used as healthy controls, and the rats were also randomly assigned either to an untreated (Control, *n* = 6) or a hyperbaric treatment (Control HB, *n* = 6) group. The rats were housed in a controlled environment with a fixed 12 h light-dark cycle (lights on from 08:00 to 20:00) and a constant temperature of 22 ± 2°C. They were fed a standard diet composed of 79 g kg^−1^ moisture, 231 g kg^−1^ protein, 51 g kg^−1^ fat, 58 g kg^−1^ ash, 28 g kg^−1^ fiber, and 553 g kg^−1^ carbohydrates. Food and water were provided *ad libitum*.

### 2.2. Hyperbaric Treatment

From ages 24–40 wks, the rats in the Diabetes HB and Control HB groups were exposed to 1.3 ATA (1317.225 hPa) for 8 h d^−1^ (23:30–07:30), 5 d wk^−1^. Hyperbaric treatment at 1.3 ATA is one of the complementary treatments at home. We are assuming the treatment will be used during sleep. Therefore, treatment time was determined for 8 h a day. The cages of the nontreated Diabetes and Control groups were placed in a hyperbaric chamber set to normal atmospheric pressure (1013.25 hPa) to equalize compressor noise. The food intake levels of both the nontreated and treated groups were equal.

### 2.3. Oral Glucose Tolerance Test

At 48 h after the final hyperbaric treatment, an oral glucose tolerance test (OGTT) was performed to determine glucose tolerance relative to body weight. The rats were fasted for 12 h, and glucose (2 g kg^−1^ body weight) was administered via an esophageal feeding tube. Blood samples were obtained from the lateral caudal vein before and at 30, 60, and 120 min after glucose administration. The blood samples were centrifuged at 3000 rpm for 10 min at room temperature, and the plasma fraction was stored at -80°C until subsequent analysis. Glucose and insulin concentrations were measured with commercially available spectrophotometric (298-65701; Wako, Osaka, Japan) and enzyme-linked immunosorbent (ELISA) (M1101; Morinaga, Yokohama, Japan) assay kits, respectively, according to the manufacturers' instructions.

As a supplementary experiment, male Sprague-Dawley rats aged 20–21 wks (*n* = 6) were used to determine the immediate effects of the hyperbaric treatment. After their fasting blood glucose levels were measured, the rats were exposed to 1.3 ATA for 60 min and their blood glucose levels were measured again.

### 2.4. Tissue Sampling

At 48 h after the OGTT, the rats were fasted for 12 h and sacrificed by sodium pentobarbital overdose. The epididymal adipose tissue, plantaris muscle, and soleus muscle were removed, weighed, frozen in liquid nitrogen, and stored at -80°C until further analysis. The tissue samples were used to study mRNA expressions and to make frozen sections for the skeletal muscles.

### 2.5. Blood Analysis

Plasma samples collected at fasting were used to determine triacylglycerol (TAG) and free fatty acid (FFA) concentrations. TAG and FFA were measured with commercially available spectrophotometric assay kits (294-63601 and 290-63701, respectively; Wako, Osaka, Japan) according to the manufacturer's instructions.

### 2.6. Histological Analysis

Portions of the epididymal adipose tissue were removed, fixed with 4% paraformaldehyde in 0.1 M phosphate buffer, and embedded in paraffin. Tissue sections were stained with hematoxylin and eosin. The diameters of ≥100 adipose cells/rat in two randomly chosen fields were measured with ImageJ (NIH, Bethesda, MD, USA).

Transverse sections of the middle part of the muscle belly in the plantaris and soleus muscles were obtained with a cryostat. The sections were stained for myofibrillar adenosine triphosphatase (ATPase, pH 4.55) activity to categorize the muscle fiber as type I, IIA, or IIB based on a previous study [16]. The cross-sectional areas of the dominant muscle fiber (type IIB and type I fibers in the plantaris and soleus muscles, respectively) were measured for three randomly chosen fields with ImageJ (NIH, Bethesda, MD, USA). For each rat, ≥100 muscle fibers were measured.

### 2.7. Quantitative Polymerase Chain Reaction (qPCR) Analysis

The tissue samples to study mRNA expressions were extracted at 48 h after OGTT. Total RNA was isolated with a TRIzol reagent (15596-026; Invitrogen, Tokyo, Japan). The total RNA concentration was determined with a spectrophotometer (NanoDrop; Thermo Fisher Scientific, Waltham, MA, USA) and calibrated to be equal for all samples. Reverse transcription was performed with a High-Capacity cDNA Reverse Transcription Kit (4374966; Applied Biosystems, Foster City, CA, USA). The cDNA samples were stored at -20°C until subsequent analysis.

The mRNA expression levels of IL-6 (Rn01410330_m1), TNF*α* (Rn01525859_g1), IL-1*β* (Rn00580432_m1), and IL-10 (Rn00563409_m1) were quantified by qPCR with TaqMan Gene Expression Assays (Applied Biosystems, Foster City, CA, USA). Relative expression levels were inferred by normalizing the quantity of the cDNA template per gene against the quantity of cDNA for the 18S (Rn03928990_g1) reference standard. The cDNA concentration at each qPCR cycle was plotted to obtain a standard curve. The relative gene expression corresponded to the value at the threshold line. The qPCR was performed with a PCR Fast Advanced Master Mix (Applied Biosystems, Foster City, CA, USA) in CFX96 (Bio-Rad Laboratories, Hercules, CA, USA).

### 2.8. Near-Infrared Spectroscopy (NIRS) Analysis of Skeletal Muscle Hemodynamic Response to Hyperbaric Treatment

Male OLETF (649 ± 18 g, *n* = 3) and LETO (546 ± 0 g, *n* = 3) rats aged 36 wks were used to assess skeletal muscle hemodynamics during hyperbaric treatment. The rats were lightly anesthetized by intraperitoneal sodium pentobarbital administration (25 mg kg^−1^). The NIRS probe was placed on the muscle belly in the gastrocnemius lateralis muscle and secured with adhesive tape. The rats were restrained with a plastic holder. Measurements began 40 min after light anesthesia induction. The concentrations of oxyhemoglobin (Oxy-Hb) and deoxyhemoglobin (Deoxy-Hb) were measured in the calf muscles 3–5 mm beneath the probe. Total hemoglobin (Total-Hb: sum of Oxy-Hb and Deoxy-Hb) and oxygen saturation (StO_2_: percentage of Oxy-Hb in Total-Hb) were calculated from these concentrations. Measurements were performed at normal atmospheric pressure (1 ATA) for 5 min, then at 1.3 ATA for 10 min. Hemodynamics was analyzed with BOM-L1TRSF (Omegawave, Tokyo, Japan).

### 2.9. Statistical Analysis

Data are means ± SEM. Significant differences between group means were identified by two-way ANOVA. In a supplementary experiment, the significant differences between nontreatment and the hyperbaric treatment were evaluated using an independent *t*-test. Statistical significance was set at *p* < 0.05. All statistical analyses were performed using SPSS statistical analysis software (IBM SPSS Statistics version 19.0; IBM Japan, Tokyo, Japan).

## 3. Results

### 3.1. Glucose and Insulin Levels during OGTT

**Figure 1:**
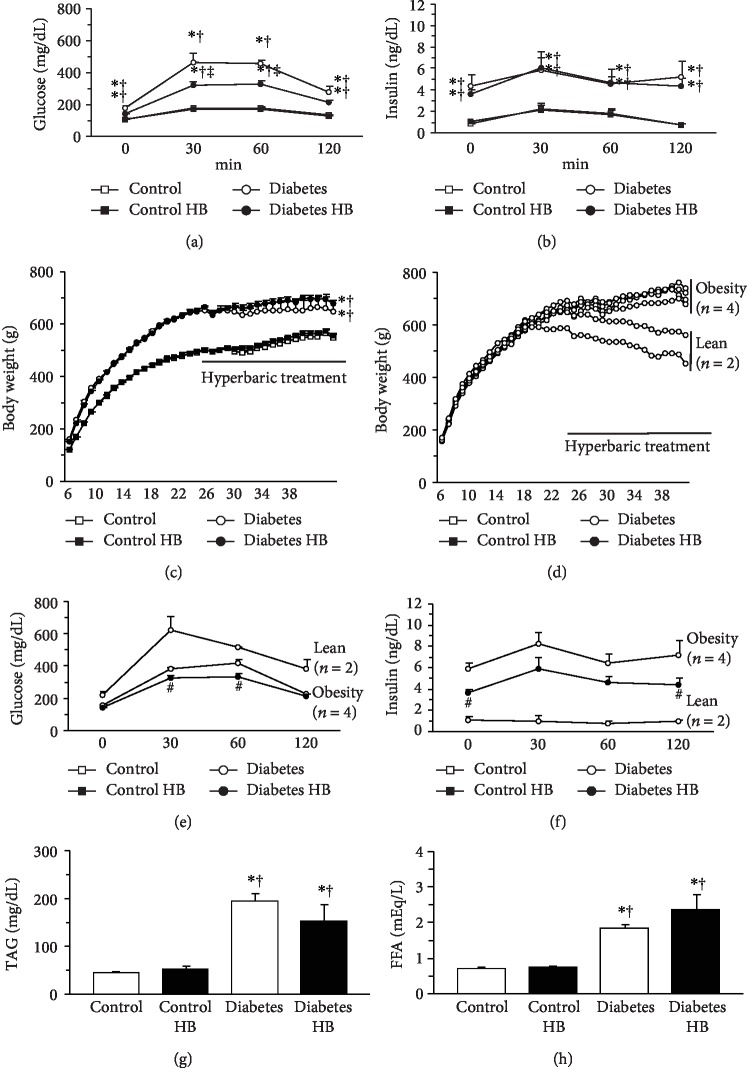
Glucose tolerance, insulin sensitivity, and metabolic properties. Glucose levels (a) and insulin levels (b) during OGTT, time course of body weight changes (c), values for the diabetes group (d–f), fasting plasma TAG levels (g), and FFA levels (h). Control: nontreated control group; Control HB: control treated with the hyperbaric normoxia group; Diabetes: nontreated diabetes group; Diabetes HB: diabetes treated with the hyperbaric normoxia group. The Diabetes group was further divided into the Diabetes obesity and Diabetes lean subgroups. Time zero: fasting. Values are means ± standard deviation. ^∗^^,†,‡^Significantly different from the Control, Control HB, and Diabetes groups, *p* < 0.05, respectively. ^#^Significantly different from the Diabetes obesity and Diabetes lean subgroups, *p* < 0.05.

Glucose levels were significantly higher in the diabetic rats than in the healthy control rats at fasting, 30, 60, and 120 min after glucose administration (Figure 1(a)). However, the glucose levels at 30 and 60 min after glucose administration were significantly lower in the Diabetes HB group than in the Diabetes group. Insulin levels were significantly higher in the diabetic rats than in the healthy control rats at fasting, 30, 60, and 120 min after glucose administration (Figure 1(b)). No significant differences in insulin level were noted between the Diabetes and Diabetes HB groups.

Throughout the experimental period, body weight was significantly higher for the diabetic rats than for the healthy control rats (Figure 1(c)). Body weight at the end of the experiment was slightly higher for the Diabetes HB group than for the Diabetes group (Diabetes, 663 ± 99 g; Diabetes HB, 697 ± 38 g). Although the body weight increased daily for four rats (Diabetes obesity subgroup) in the Diabetes group, the body weights of two rats (Diabetes lean subgroup) in the Diabetes group gradually decreased after the middle of the experimental period (Figure 1(d)). The mean body weights at the end of the experiment were 729 ± 23 g and 530 ± 42 g in the obese and lean subgroups of the Diabetes group, respectively. The body weight increased daily for all rats in the Diabetes HB group. The glucose levels were always higher in the Diabetes lean than in the Diabetes obese subgroup (Figure 1(e)). Conversely, the insulin levels were always lower in the Diabetes lean than in the Diabetes obese subgroup (Figure 1(f)). The insulin levels at fasting and 120 min after glucose administration were significantly lower in the Diabetes HB group than in the obesity subgroup of the Diabetes group.

Fasting plasma TAG (Figure 1(g)) and FFA (Figure 1(h)) levels were significantly higher in the diabetic than in the healthy control rats. No significant differences in the TAG and FFA levels were noted between the Diabetes and Diabetes HB groups.

### 3.2. Glucose Levels Immediately after Hyperbaric Treatment

A supplementary experiment revealed that neither nontreatment nor the hyperbaric treatment decreased the glucose levels immediately following glucose administration. In the absence of hyperbaric treatment, the mean glucose levels at fasting and at 60 min after glucose administration were 110 ± 9 mg dL^-l^ and 154 ± 11 mg dL^-l^, respectively. With hyperbaric treatment, the mean glucose levels at fasting and at 60 min after glucose administration were 116 ± 6 mg^-l^ and 156 ± 13 mg^-l^, respectively. There was no significant difference between the rats with and without hyperbaric treatment in terms of glucose level at 60 min after glucose administration.

### 3.3. Epididymal Adipose Tissue

**Figure 2:**
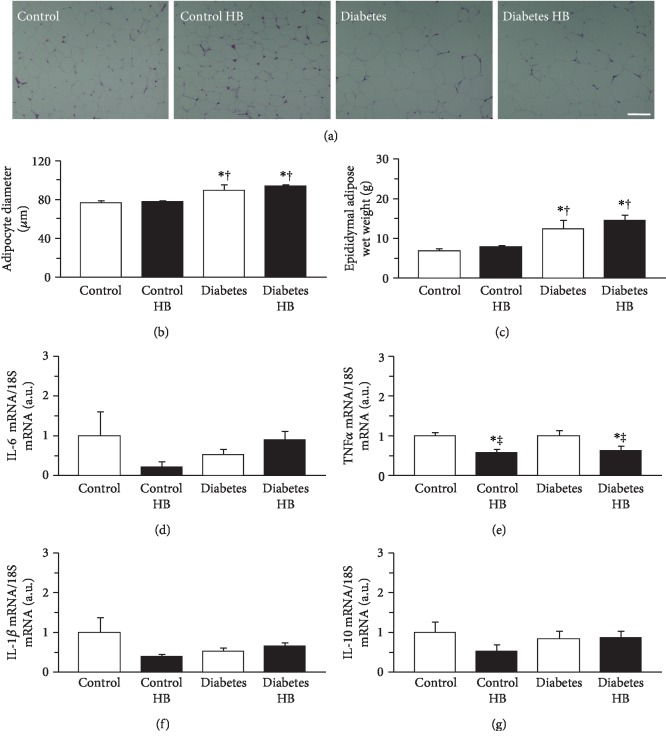
Adipocyte size, adipose tissue mass, and cytokine expression in adipose tissue. Representative sections of epididymal adipose tissue stained with hematoxylin and eosin (a), adipocyte diameter (b), wet weight of adipose tissue (c), expression levels of IL-6 mRNA (d), TNF*α* mRNA (e), IL-1*β* mRNA (f), and IL-10 mRNA (g). Control: nontreated control group; Control HB: control treated with the hyperbaric normoxia group; Diabetes: nontreated diabetes group; Diabetes HB: diabetes treated with the hyperbaric normoxia group. Bar = 100 *μ*m. Values are means ± standard deviation. IL-6, TNF*α*, IL-1*β*, and IL-10 mRNA expression levels were calculated as fold changes relative to the Control group. ^∗^^,†,‡^Significantly different from the Control, Control HB, and Diabetes groups, *p* < 0.05, respectively.

Adipocyte enlargement was more frequently observed in the diabetic than in the healthy control rats. However, hypertrophic adipocytes > 130 *μ*m in diameter were rare in both cases (Figure 2(a)). Adipocyte diameters were significantly larger in the diabetic than in the healthy control rats (Figure 2(b)). The mean adipocyte diameter in the Diabetes group was 1.16x greater than that in the Control group. There was no significant difference between the Diabetes and Diabetes HB groups in terms of adipocyte diameter. The wet weight of epididymal adipose tissue was significantly greater in the diabetic than in the healthy control rats (Figure 2(c)). The mean epididymal adipose tissue weight in the Diabetes group was 1.78x larger than that in the Control group. There was no significant difference between the Diabetes and Diabetes HB groups in terms of the wet weight of epididymal adipose tissue.

The expression levels of IL-6, TNF*α*, IL-1*β*, and IL-10 mRNA are shown in Figures 2(d)–2(g), respectively. There were no significant differences among groups in terms of the expression levels of IL-6, IL-1*β*, and IL-10 mRNA. On the other hand, the expression levels of TNF*α* were significantly lower in the rats exposed to the hyperbaric environment than in those not exposed to it (Figure 2(e)).

### 3.4. Skeletal Muscle

**Figure 3:**
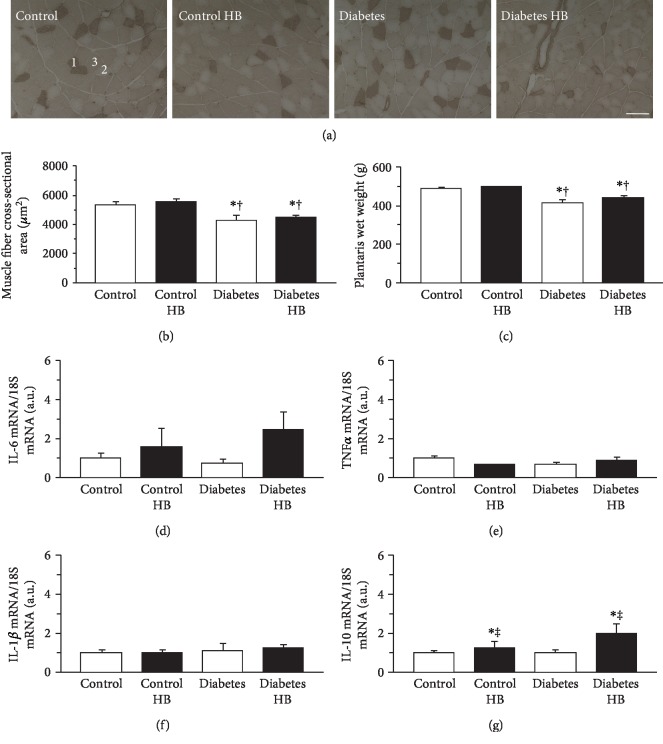
Muscle fiber cross-sectional area, wet weight, and cytokine expression in the plantaris muscle. Representative sections of the plantaris muscle stained for ATPase activity (a), type IIB fiber cross-sectional area (b), wet weight (c), expression levels of IL-6 mRNA (d), TNF*α* mRNA (e), IL-1*β* mRNA (f), and IL-10 mRNA (g). Control: nontreated control group; Control HB: control treated with the hyperbaric normoxia group; Diabetes: nontreated diabetes group; Diabetes HB: diabetes treated with the hyperbaric normoxia group; 1: type I fiber; 2: type IIA fiber; 3: type IIB fiber. Bar = 100 *μ*m. Values are means ± standard deviation. IL-6, TNF*α*, IL-1*β*, and IL-10 mRNA expression levels were calculated as fold changes relative to the Control group. ^∗^^,†,‡^Significantly different from the Control, Control HB, and Diabetes groups, *p* < 0.05, respectively.

ATPase staining revealed that the plantaris muscles were composed of type I, IIA, and IIB fibers (Figure 3(a)). Type IIB fiber predominated in all groups. The cross-sectional area of the type IIB fiber in the diabetic rats was significantly smaller than that in the healthy control rats (Figure 3(b)). There was no significant difference between the Diabetes and Diabetes HB groups in terms of the cross-sectional area of the type IIB fiber. The wet weight of the plantaris muscle was significantly lower in the diabetic than in the healthy control rats (Figure 3(c)). There was no significant difference between the Diabetes and Diabetes HB groups in terms of the wet weight of the plantaris muscle.

The expression levels of IL-6, TNF*α*, IL-1*β*, and IL-10 mRNA in the plantaris muscle are shown in Figures 3(d)–3(g), respectively. There were no significant differences among groups in terms of the expression levels of IL-6, TNF*α*, and IL-1*β* mRNA. Nevertheless, the expression levels of IL-10 were significantly higher in rats exposed to the hyperbaric environment than in those not exposed to it (Figure 3(g)).

**Figure 4:**
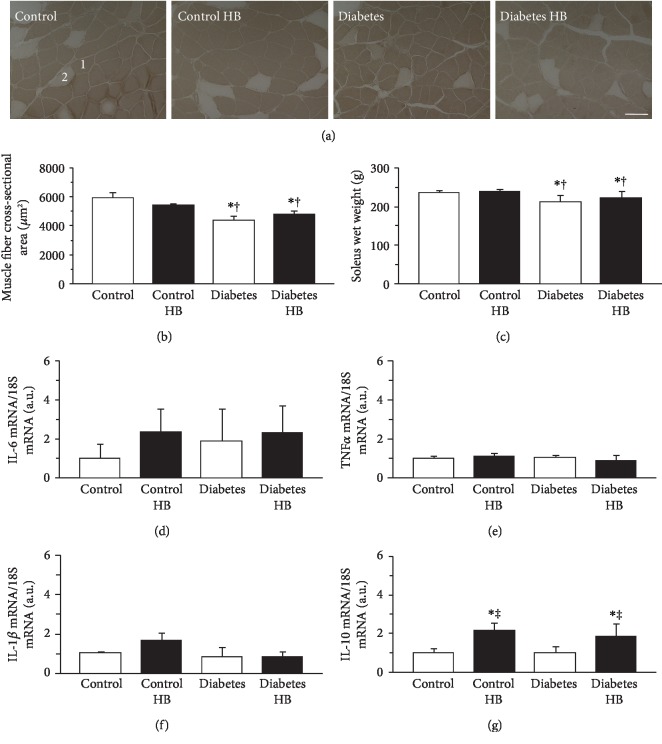
Muscle fiber cross-sectional area, wet weight, and cytokine expression in the soleus muscle. Representative sections of the soleus muscle stained for ATPase activity (a), type I fiber cross-sectional area (b), wet weight (c), expression levels of IL-6 mRNA (d), TNF*α* mRNA (e), IL-1*β* mRNA (f), and IL-10 mRNA (g). Control: nontreated control group; Control HB: control treated with the hyperbaric normoxia group; Diabetes: nontreated diabetes group; Diabetes HB: diabetes treated with the hyperbaric normoxia group; 1: type I fiber; 2: type IIA fiber. Bar = 100 *μ*m. Values are means ± standard deviation. IL-6, TNF*α*, IL-1*β*, and IL-10 mRNA expression levels were calculated as fold changes relative to the Control group. ^∗^^,†,‡^Significantly different from the Control, Control HB, and Diabetes groups, *p* < 0.05, respectively.

The soleus muscles were composed of type I and IIA fibers (Figure 4(a)) with the type I fiber predominating in all groups. The muscle fiber cross-sectional area and the wet weight of the soleus muscle resembled those of the plantaris muscle. The cross-sectional area of the type I fiber (Figure 4(b)) and the wet weight of the soleus muscle (Figure 4(c)) were significantly smaller in the diabetic than in the healthy control rats. There were no significant differences between the Diabetes and Diabetes HB groups in terms of the cross-sectional area of the type I fiber and the wet weight of the soleus muscle.

The expression levels of IL-6, TNF*α*, IL-1*β*, and IL-10 mRNA in the soleus muscle are shown in Figures 4(d)–4(g), respectively. There were no significant differences among groups in terms of the expression levels of IL-6, TNF*α*, and IL-1*β* mRNA. However, the expression levels of IL-10 were significantly higher in rats exposed to the hyperbaric environment than in those not exposed to it (Figure 4(g)).

### 3.5. Skeletal Muscle Hemodynamic Response to Hyperbaric Treatment

**Figure 5:**
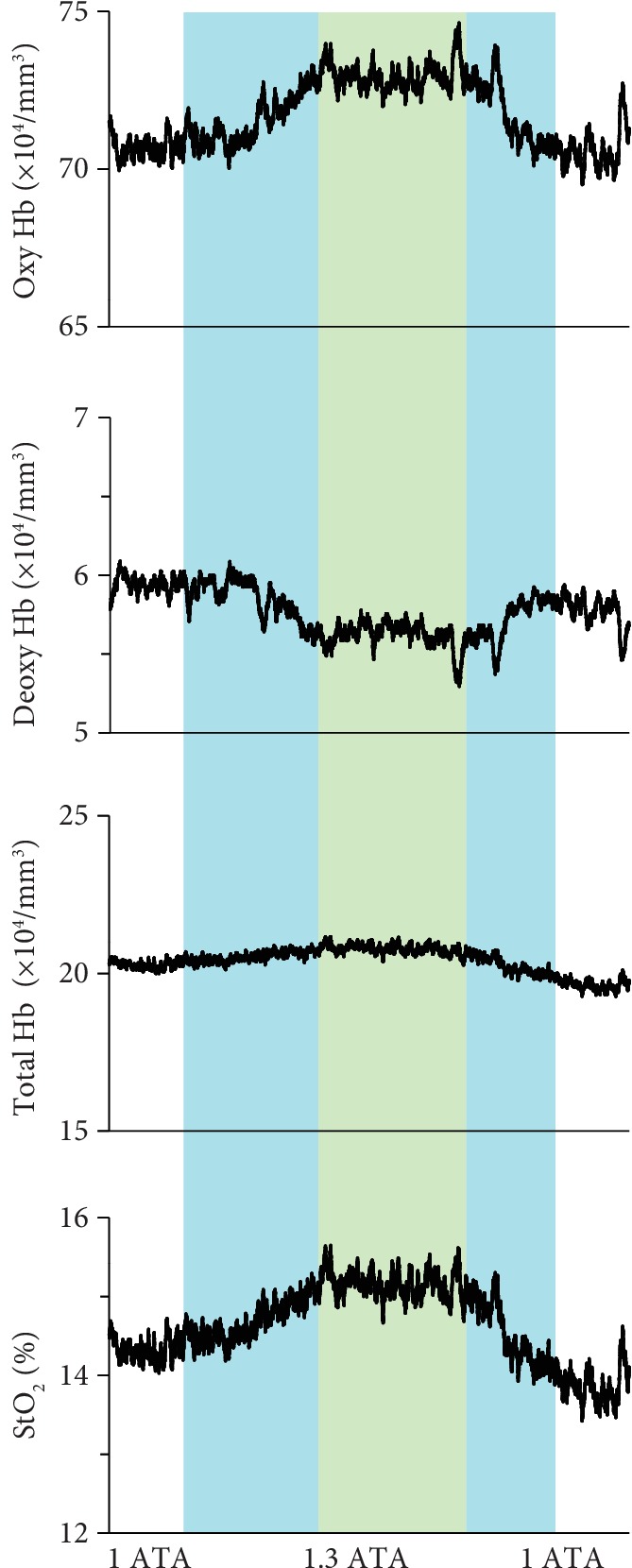
Representative NIRS data for calf muscles during hyperbaric treatment. The green area indicates 1.3 ATA, and blue areas indicate early compression and late decompression periods. Each variable was sequentially calculated every 0.1 s.

**Figure 6:**
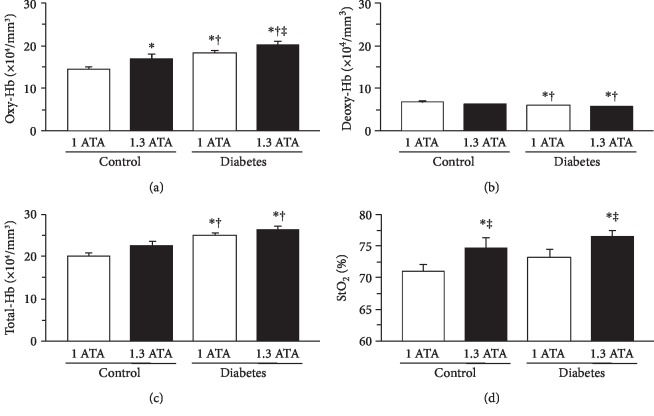
Skeletal muscle hemodynamic response to hyperbaric treatment. Oxy-Hb (a), Deoxy-Hb (b), Total-Hb (c), and StO_2_ (d). Values are means ± standard deviation. ^∗^^,†,‡^Significantly different from the Control, Control HB, and Diabetes groups, *p* < 0.05, respectively.

Hyperbaric treatment substantially elevated Oxy-Hb, slightly reduced Deoxy-Hb, and increased StO_2_ in the calf muscles (Figure 5). The mean Oxy-Hb was significantly higher at 1.3 ATA than at 1.0 ATA (Figure 6(a)). There were no significant differences between the 1.0 ATA and 1.3 ATA treatments in terms of the mean Deoxy-Hb (Figure 6(b)) or mean Total-Hb (Figure 6(c)). The mean StO_2_ was significantly higher at 1.3 ATA than at 1.0 ATA (Figure 6(d)).

## 4. Discussion

In the present study, regular hyperbaric normoxia at 1.3 ATA improved glucose levels at 30 and 60 min after glucose administration and insulin levels at fasting and 120 min after glucose administration in a rat model of type 2 diabetes with obesity. It also upregulated IL-10 in the skeletal muscle and downregulated TNF*α* in adipose tissue.

OLETF rats are missing the satiety signal receptor in the ventromedial nucleus of the hypothalamus. Consequently, they develop hyperphagia and eventually become obese [17]. Obesity is associated with increased expression of proinflammatory cytokines such as IL-6, TNF*α*, and IL-1*β* in adipose tissue. These factors have detrimental effects on the insulin signaling pathway [18]. Obesity-related insulin resistance and glucose intolerance have been attributed to defects in the insulin signaling pathway [19]. In the present study, there were no significant differences between the diabetic and healthy control rats in terms of the expression levels of IL-6, TNF*α*, and IL-1*β* mRNA per unit adipose tissue mass. Proinflammatory cytokines are secreted by adipocytes and macrophages in adipose tissue [1, 20]. Hypertrophic adipocytes [21] and macrophages shifted the polarization from the M2 to the M1 phenotype [22] frequently secrete TNF*α* and other cytokines. In the present study, hypertrophic adipocytes with diameters > 130 *μ*m were seldom observed in diabetic rats. The relative lack of large hypertrophic adipocytes may partially account for the fact that there was no significant difference in adipose TNF*α* expression between the diabetic and healthy control rats. However, both adipocyte size and tissue mass were significantly larger in the diabetic than in the healthy control rats. Thus, the diabetic rats could be more strongly affected by proinflammatory cytokines than the healthy control rats.

Previous studies reported that hyperbaric treatment improves hyperglycemia and hyperinsulinemia in both obese [13] and nonobese type 2 diabetic animals [14, 15]. Nevertheless, the mechanism for this improvement remains unknown. In the present study, regular hyperbaric normoxia at 1.3 ATA improved glucose and insulin levels after glucose administration in diabetic rats. No progressive weight loss, hyperglycemia, or hypoinsulinemia [23] was detected in obese type 2 diabetic rats treated with 1.3 ATA. The present study revealed that the efficacy of hyperbaric treatment is associated with improvements in insulin sensitivity and glucose tolerance, downregulation of TNF*α* expression in adipose tissue, and upregulation of IL-10 in the skeletal muscle. It is widely accepted that IL-10 inhibits proinflammatory cytokine signaling induced by TNF*α* and others [24]. IL-10 also has efficacy in insulin sensitivity and glucose tolerance in type 2 diabetes with obesity. Kim et al. reported that acute IL-10 treatment improved whole-body insulin action and glucose metabolism during a hyperinsulinemic-euglycemic clamp [25]. Bhargava et al. reported that improvements in insulin sensitivity and glucose tolerance in obese mice were mediated by increased IL-10 production [26]. Grant et al. reported that IL-10 upregulation with concomitant TNF*α* downregulation improved insulin sensitivity and glucose tolerance in obese mice [27]. IL-10 is expressed and released by the skeletal muscle [28]. It is upregulated during physical exercise [29]. In addition to physical exercise, hyperbaric treatment increases IL-10 expression in injured skeletal muscles [30]. In health, IL-10 originating from the skeletal muscle interacts with adipose and other tissues [31]. Therefore, decreased TNF*α* expression and increased IL-10 expression in diabetic rats under hyperbaric treatment participate in the crosstalk between the adipose tissue and skeletal muscle. Increased tissue oxygenation by hyperbaric treatment could promote the production of anti-inflammatory IL-10 in the skeletal muscles and decrease the production of proinflammatory TNF*α* in the adipose tissues through inhibition of nuclear factor-kappa B. However, the reasons that IL-10 was increased by hyperbaric treatment were found not in the adipose tissue but in the skeletal muscles; also, as to why IL-6 and IL-1*β* were not influenced by the treatment is still unclear in the present study.

Regular hyperbaric treatment improved glucose and insulin levels after glucose administration in diabetic rats. Nevertheless, it had no efficacy for obesity or hyperlipidemia. Moreover, no acute reduction in the glucose level was observed after hyperbaric treatment in the supplementary experiment. Thus, hyperbaric treatment does not promote glucose consumption or inhibit lipid accumulation; rather, it accelerates glucose uptake.

In the present study, skeletal muscle mass and fiber size were significantly smaller in the diabetic than in the healthy control rats. Hyperbaric treatment did not have any influence on the skeletal muscle mass defects in diabetic rats. Skeletal muscle atrophy usually occurs in type 2 diabetes and is controlled by the action of TNF*α* on the protein degradation pathway [32]. Conversely, TNF*α* downregulation in the skeletal muscle inhibits protein degradation [33]. In the present study, there was no significant difference among all groups in terms of the TNF*α* expression level in both the plantaris and soleus muscles. In a preliminary study, there was no significant difference between the OLETF and LETO rats in terms of the muscle RING finger 1 (E3 ubiquitin ligase) expression level (data not shown). Our previous study [11] showed that the skeletal muscle mass is nearly the same at 24 and 40 wks in OLETF rats (~200 g in the soleus muscle). Therefore, structural protein degradation did not occur during this time interval. Skeletal muscle atrophy in type 2 diabetes is associated with physical inactivity, reduced food intake, and peripheral neuropathy. Therefore, the defects in the skeletal muscle mass of OLETF rats may have been caused by the downregulation of protein synthesis which may occur as a result of physical inactivity [34, 35], immobilization [36, 37], denervation [38], and cachexia [39]. Hyperbaric treatment does not seem to enhance structural protein synthesis in the skeletal muscle.

The present study revealed that hyperbaric treatment at 1.3 ATA upregulates IL-10 in the skeletal muscle and improves insulin sensitivity and glucose tolerance in type 2 diabetes with obesity. Nevertheless, this study has certain limitations. The mechanism by which hyperbaric treatment upregulates IL-10 in the skeletal muscle has not been elucidated. It was proposed that obesity causes chronic hypoxia and hypoxia-inducible dysfunction which, in turn, contribute to insulin resistance and glucose intolerance [40]. Therefore, tissue oxygenation during hyperbaric treatment may reverse hypoxia-inducible dysfunction. Hypoxia and hypoxia-related genes increased in the adipose tissue of obese animals. In contrast, no hypoxia-related gene upregulation was detected in the skeletal muscle [41]. In the present study, there was no significant difference between diabetic and healthy control rats in terms of StO_2_ in their calf muscles. Although hypoxia was not observed in the skeletal muscle, hyperbaric treatment increased oxygenation which, in turn, upregulated IL-10 in this tissue. Therefore, several factors may be involved in IL-10 upregulation in the skeletal muscle. Certain cytokines derived from skeletal muscle fibers may affect adipocytes and improve glucose tolerance [42]. In the present study, however, it was unclear whether IL-10 originating in the skeletal muscle has an endocrine-like impact on TNF*α* expression in adipose tissue. Further research is required to elucidate the mode of action of hyperbaric treatment in type 2 diabetes with obesity. Additionally, the study to find out the optimum intervention term is also needed to generalize the treatment in the patients.

## 5. Conclusion

Regular hyperbaric treatment with normal air at 1.3 ATA significantly lowered blood glucose and insulin levels, upregulated anti-inflammatory IL-10 in the skeletal muscle, and downregulated proinflammatory TNF*α* in adipose tissue of rats with obese type 2 diabetes compared to untreated rats with the same condition. Hyperbaric treatment did not promote glucose consumption, inhibit lipid accumulation, or enhance structural protein synthesis in the skeletal muscle. However, hyperbaric treatment would rather accelerate glucose uptake.

## Data Availability

The data that support the findings of this study are available from the corresponding author upon reasonable request.
